# The Code of Practice and its enduring relevance in Europe and Eastern and Southern Africa

**DOI:** 10.1186/s12960-016-0122-y

**Published:** 2016-06-30

**Authors:** Remco van de Pas, Linda Mans, Giulia de Ponte, Yoswa Dambisya

**Affiliations:** Health Policy Unit, Department of Public Health, Institute of Tropical Medicine, Nationalestraat 155, 2000 Antwerp, Belgium; Wemos Foundation, P.O. Box 1693, 1114 AK Amsterdam, The Netherlands; Amref Health Africa Onlus, via Alberico II, n.4, 00193 Rome, Italy; ECSA Health Community Secretariat, 157 Olorien, Njiro Road, P.O. Box 1009 Arusha, Tanzania

**Keywords:** Civil society, Code of Practice, Governance, Migration, Recruitment, World Health Organization

## Abstract

**Background:**

The relevance and effectiveness of the WHO Global Code of Practice on the International Recruitment of Personnel will be reviewed by the World Health Assembly in 2015. The origins of the Code of Practice and the global health diplomacy process before and after its adoption are analyzed herein.

**Methods and Results:**

Case studies from the European and eastern and southern African regions describe in detail successes and failures of the policy implementation of the Code. In Europe, the Code is effective and even more relevant than before, but might require some tweaking. In Eastern and Southern Africa, the code is relevant but far from efficient in mitigating the negative effects of health workforce migration.

**Conclusions:**

Solutions to strengthen the Code include clarification of some of its definitions and articles, inclusion of a governance structure and asustainable and binding financing system to reimburse countries for health workforce losses due to migration, and featuring of health worker migration on global policy agendas across a range of institutional policy domains.

## Background

### The origins of the Code of Practice

The recruitment of health workers from abroad is part of an expansive pattern of skilled workforce migration that has existed since the rapid welfare state expansion of many countries in the 1950s and 1960s. Already in 1972, 6 % of the world’s physicians were located outside their country of origin [1]. The development of the World Health Organization (WHO) Global Code of Practice on the International Recruitment of Health Personnel (referred to as the ‘Code’ henceforth) was preceded by bilateral codes that aimed at mitigating the migration of health workers to richer countries. For instance, the United Kingdom Department of Health introduced a Code of Practice for international recruitment for National Health Service employers in 2001 [2]. A study that assessed the relevance of this code concluded that it was difficult to evaluate its actual impact due to a limited monitoring capacity, a multiplicity of factors besides active recruitment that influence the mobility of the workforce, and the limited visibility of this code in source countries [3]. Other voluntary codes of practice and similar non-binding instruments have been widely criticized as weak and ineffective in mitigating workforce imbalances related to the migration of health workers [4]. Despite this criticism, in 2004, the World Health Assembly mandated the Director General to develop a non-binding code of practice on the international recruitment of health personnel [5]. Simultaneously, the Joint Learning Initiative on Human Resources for Health and Development called for mobilizing and strengthening human resources for health (HRH) as a key strategy to combat the health crises in the world’s poorest countries and to build sustainable health systems everywhere [6]. In order to cope with the health workforce crisis, the Joint Learning Initiative report proposed that effective country strategies should be reinforced internationally, “*Ultimately, the crisis in human resources is a shared problem requiring shared responsibility for cooperative action*” [6]. This agenda was enforced with the release of the World Health Report 2006, *Working Together for Health* [7], and the creation of the Global Health Workforce Alliance (GHWA) in 2006. A decade of action on HRH thus commenced.

### A lost investment

In an interconnected world, globalization and scarcity are closely linked. The fiscal realities that frame available public financing for health systems and health workforce salaries are shaped by such issues as untaxed wealth, capital flight, wealth inequalities, etc. This fiscal crisis (including former ‘ceilings’ on expenditure of the health workforce public wage bill, imposed by the International Monetary Fund in a number of African countries until 2007) has contributed to external migration, which, in turn, has caused significant savings in training costs to importing countries [8].

In nine African source countries, the estimated government-subsidized cost of a doctor’s education ranges from US$ 21,000 in Uganda to US$ 58,700 in South Africa. The overall estimated loss of return on investment for all doctors currently working abroad is US$ 2.17 billion, ranging from US$ 2.16 million for Malawi to US$ 1.41 billion for South Africa. The benefit to destination countries of recruiting trained doctors was largest for the United Kingdom (US$ 2.7 billion) and the United States (US$ 846 million) [9]. As a counter-argument Clemens reasons that many countries in the African region simply lack the absorption capacity to integrate the workforce either in the public or private sector [10]. Migrated African physicians in the United States and Canada send, on average, more than $ 4,500 per year to their countries of birth; these remittances will be used by private actors within the country and are higher than what leaves the public coffers [10]. However, several countries, especially those from the WHO African Region, when discussing the second draft of the Code during WHO’s Executive Board meeting in January 2009, expressed the view that it needed more ‘teeth’ for enforcement and advised that it should include mechanisms to compensate developing countries for the migration to higher income countries [11]. High-income countries, especially the United States, recommended not to link the provision of development assistance to recruiting practices. During the drafting and consultations on the Code that lasted from 2008 to 2010, the Health Worker Migration Initiative, a partnership of Realizing Rights (the ethical globalization initiative chaired by Mary Robinson), GHWA, and WHO, facilitated the negotiations. This included the commissioning of a paper on potential strengths of non-binding instruments in international legal practice. The Health Worker Migration Initiative, together with Norway and the WHO Regional Office for Europe, also convened an inter-regional dialogue in Madrid in May 2010 to allow participants to get acquainted with the text and discuss content issues. The Code’s non-binding character is considered as an advantage, as it allows flexibility, including with regards to future adaptation. The code sets forth a “*deep legal and institutional framework*” and may “*promote deeper commitments*” than legally binding instruments [11]. A week later, the Code was adopted at the sixty-third World Health Assembly, slightly modified though as high-income countries argued that the tone was too prescriptive or mandatory for a non-binding instrument – this modification has perhaps softened the sense of obligations amongst countries to comply with the different articles of the code [11]. The Code focuses on ethical international recruitment and fair treatment of migrant health workers, but also includes statements on self-sustainability in national health workforces, international cooperation, support to developing countries, data gathering, and information exchange. Therefore, it comprehensively lays the ground for engagement on several aspects of the health workforce, especially in developing countries.

WHO recommended that the Code be incorporated into national policies and laws so that it can become legally binding. However, some states suggested that a more formal system for monitoring and implementing the Code was necessary for it to become a meaningful response to global HRH recruitment. The adoption of the Code, unfortunately, marked the end of a few ‘good years for HRH’ in global health policy. The economic crises in the United States and Europe led to a reduction of funds for GHWA and WHO to effectively work on Code implementation and monitoring. Austerity in Europe and the United States put a strain on health systems, including the health workforce [9]. Despite these resource constraints, a small but dedicated group of actors from different organizations and countries have been actively involved in the Code follow-up and implementation in recent years.

The health workforce crisis should not be dealt with within its own thematic ‘silo’ , but should rather be looked at in a systemic way. The global health workforce gap has increased rather than decreased since the release of the World Health Report in 2006. Given current population growth rates in different regions in the world, an ageing workforce, and an epidemiological transition to chronic disease worldwide, there is a desperate need for more skilled health workers. In 2013, approximately 7.2 million more midwives, nurses, and physicians were “*missing and thus not in action*” – and this shortfall is predicted to rise further to at least 12.9 million in the coming decade [12]. The Ebola outbreak in West Africa in 2014 indicated how vulnerable health systems really are when a skilled workforce with core capacities for epidemic response is missing. The outbreak was yet another wake-up call for the international community and national governments to develop the global health workforce urgently [13].

## Methods and Results

Against this backdrop, the relevance and effectiveness of the Code has been assessed in a number of European countries and in Eastern and Southern Africa (ESA). These regions were selected for the present review given that the authors, active in academia and civil society, have been closely involved in Code follow-up and policy dialogue over the last few years. In this analysis, the authors provide their experiences with and insights into the uptake of the Code and its potential for future directions.

### The relevance of the Code of Practice in Europe

The period of implementation of the Code in the European region has coincided with the financial and economic crisis. The latter impacts directly on the relationship between investment in health workforce development and health workforce mobility, which is at the heart of the Code: the resulting new intra-European Union (EU) wage imbalances and the persisting shortages of health workers confer to the Code a renewed relevance in the region.

Although countries in Europe have responded to the economic crisis in various ways, most have adopted large-scale cuts and public sector reforms: in the context of the austerity packages implemented in 2009–2011, public spending on health fell in many countries [14]. As health worker costs account for the largest share of spending on health, these costs have been a common target for budget cuts, also in countries where salaries are relatively low [15].^1^ Wage imbalances between countries (depending on changes in wages in immigration countries compared to emigration countries) or within countries (if the private and public sector have different rates of pay) are therefore changing considerably and have the potential to increase health workforce mobility in the region and beyond [16]. This new trend comes on top of already existing shortages: in 2012, the European Commission predicted in its *Staff Working Document on an Action Plan for the EU Health Workforce*, a potential shortfall of around 1 million healthcare workers by 2020, if no further measures were taken to meet existing challenges [17].

The response coming from EU institutions adopts a perspective that stems from considerations on the employment potential of the health sector.^2^ Health care is identified in the *Action Plan for the EU Health Workforce* as a highly labour-intensive sector [17]. As such, it is given a role in stimulating ‘a job-rich recovery’ from the economic crisis. Along the same lines, mobility of health personnel within the EU is facilitated,^3^ as the assumption is that the EU Single Market functions as a mechanism to distribute health workers to where they are most needed [18].

Using this frame, public health considerations thus tend to take second place to market development approaches. The evidence shows, however, that the free movement of health workers leads to some seeking better opportunities abroad, creating a conflict in which personal and professional ethics sometimes collide [19] at the expense of an equitable distribution of health workers in the region and beyond. This is not entirely consistent with the principles of the EU’s own Health Strategy and with the Health Programme 2014–2020, which assigns an important role to the reduction of health inequalities in the region.

The Code can be a key tool to solve this incoherence, as it brings back a much needed public health perspective into the debate on the mobility of health workers by looking at the impact, in terms of brain drain, on health systems of origin. While the value of the Code as a policy framework to manage health workforce mobility is formally acknowledged in several EU level policy documents [20], its voluntary nature implies that bold steps are yet to be taken to integrate its principles into the functioning of the Single Market: this can be done through a system of incentives and retention measures in countries of origin, and specifically by orienting EU Cohesion policy – which shapes the programming and deployment of Structural Funds – with a view to increasing support for the equitable internal distribution of a skilled health workforce.

### Practices of Code implementation in Europe: the role of non-governmental actors

In the above context, non-governmental actors, including health professionals’ organizations, trade unions, non-governmental organisations, and universities, are autonomously taking steps to implement the public health approach to health workforce mobility promoted by the Code. Civil society organisations in eight European countries^4^ have been involved in documenting these efforts as a further indication of the relevance of the Code to actors on the ground. A selection of case studies, looking at both national and local levels, is briefly presented below. The case studies focus on key areas such as ‘mobility, migration, recruitment’ , ‘planning and forecasting’ , ‘rights, working conditions, protection’ , and ‘coherence, collaboration, solidarity’.

As the labour market becomes more globalized, rising demand is driving migration and mobility amongst health personnel.

- In the Netherlands, Wemos observed that hiring cheap personnel from other European countries or even from other continents is becoming an attractive option, both for home care provided via municipalities and for private (24-h) home-based care. Different civil society organisations and trade unions are seeking collaboration between recruitment agencies, Dutch inspectorates, the Ministry of Health, the Ministry of Social Affairs and Employment, municipalities, and other trade unions in order to ensure fair recruitment and the rights of international health workers [21].

Planning, forecasting, and providing for domestic health workforces without resorting to international recruitment are key to the development of sustainable health workforces globally and a fundamental step towards reducing brain drain. This also requires reliable data about inflow and outflow of health personnel.

- In the United Kingdom, Health Poverty Action showed the engagement of the United Kingdom Royal College of Nursing in overcoming data limitations through the production of a Labour Market Review, which provides an annual picture of the United Kingdom nursing labour market, including the number of internationally recruited nurses and the wider global implications [22].
- Redemptoris Missio documented how the National Chamber of Nurses and Midwives in Poland attempted to determine the actual scale of migration using direct requests to the appropriate authorities (mainly professional associations) in other European Member States [23].

The Code extensively covers the promotion of (and respect for) fair labour practices as well as the provision of equal rights to all health personnel. Several case studies show that there are barriers, but also identify solutions.

- In Germany, Terre des Hommes analysed the nurses’ struggle for decent work at the Charité University Clinics in Berlin – a renewed trend to recruit non-European candidates was observed, unfortunately occurring at the expense of improving conditions for the nurses already in the system. Thus, the recruitment of Asian or African nurses is the result of decreasing working conditions and may act as another ‘push’ for further cuts in wages and labour rights in the German nursing sector [24].
- Terre des Hommes further analysed the German–Philippine bilateral agreement for the recruitment of nurses, finding that the inclusion of social partners in both origin and destination countries at the right time, including in the monitoring of the agreement, allowed to shape a comprehensive agreement and avert detrimental consequences [25].
- Another case study documented how increased collaboration between the European Federation of Public Service Unions, Verdi, and the Spanish Trade Unions for Health Workers (FES-CCOO and FSP-UGT) raised awareness that exploitative working conditions experienced by a group of Spanish nurses in Germany are unacceptable and that collective agreements must be respected [26].
- In the Italian province of Florence, Amref documented how IPASVI, the professional federation of nurses, put in place the first Contact Point for international health workers: it supports and helps international colleagues find their way, addressing their concerns and concrete problems such as the recognition of professional qualifications, contract, and working conditions, as well as other general living and employment issues [27].

Contributions from Europe towards achieving a sustainable health workforce and strengthening health systems worldwide require cooperation amongst several actors and a more common understanding and awareness – from global to local.

- In Belgium, the civil society-led platform for international health “Be-cause Health” engaged key actors, including the Belgian Technical Cooperation, non-governmental organisations, academic institutions, and private companies, on the issue of recruitment of foreign medical personnel, with the aim to harmonize, increase efficiency, and render more equitable the practices of Belgian development cooperation actors in this field [28].
- Memisa’s hospital twinning program stimulates professional development and exchanges between hospitals in Belgium and those in selected African countries [29].
- Amref documented how a multi-stakeholder dialogue could effectively strengthen the role of the Italian National Professional Organization of Medical Doctors (FNOMCeO) in global health, based on principles of inclusiveness and solidarity [30].
- Wemos demonstrated the role that health providers can take, through their Corporate Social Responsibility policies, in translating a global and European code at the local level in the Netherlands; this also needs various actors such as civil society organisations, trade unions, health care institutions, and recruitment agencies to help collectively raise awareness on this issue [31].
- The Center for Health Politics and Services illustrated the case of Bulgarian specialist doctors being hired part-time in the neighbouring Călăraşi region of Romania, thus ‘topping up’ their Bulgarian salaries and in this way remaining in their region without having to migrate to another EU country [32].

These case studies indicate that the public health approach to health workforce mobility promoted by the Code is already translated into practice in many local and national contexts, thanks to the efforts by a variety of non-governmental actors. They are also a confirmation that the multi-stakeholder approach promoted by the Code is key to its successful implementation. These efforts, however, are often fragmented – it is time for a more systemic approach.

As a contribution towards this end, the civil society-led *Call to Action: A Health Worker for Everyone, Everywhere* [33] was launched in 2014: it is currently gaining support at EU level, with more than 60 institutional endorsements indicating that there is a constituency of actors across Europe demanding Code implementation. The Call provides recommendations to EU institutions and Member States for strong health workforces and sustainable health systems around the world.

### Code implementation in Eastern and Southern Africa (ESA)

A study in the ESA region, with 10 countries in the region represented, found that 3 years after the Code was adopted by the World Health Assembly, the main HRH concerns in the region were considered to be internal migration, maldistribution, and absolute shortages of health professionals, rather than external migration [34]. Regarding the content of the Code, there was a perception among stakeholders that African policy interests in the negotiations on compensation and mutuality of benefits were not adequately covered in the final Code, and there were concerns regarding its voluntary nature. According to the research, Code implementation was lacking in all countries in the region, dissemination of the Code had not materialized in the region, and only one country had a designated authority. Barriers to Code implementation included lack of champions/designated authorities, poor preparedness, weak mobilisation of stakeholders, and low involvement of civil society.

The Code has not realised its potential to galvanise action on HRH in the ESA region, and yet it is one of the regions most affected by the HRH crisis. For instance, the topics of policy focus alluded to in the Code include improving migration monitoring (e.g., through a minimum core data set), managing migration flows (for instance, through bilateral agreements, memoranda of understanding, guidelines), HRH policy and practice (covering areas such as protection of the rights of migrants, promotion of circular migration, incentives for retention, better working conditions), strengthening health systems (through approaches such as health workforce planning, education, retention strategies), and coordination, collaboration, and monitoring progress. Clearly, most of the strategies needed to combat the health workforce challenges in the region can be adequately addressed through implementation of the Code.

It goes without saying that the Code is relevant in driving forward the HRH agenda, and yet there has not been much progress in implementing the Code in the ESA region since it was adopted in 2010; most progress in implementation took place in European/Organisation for Economic Co-operation and Development countries [35]. Challenges cited in the ESA region include lack of country champions, little effort by regional organisations and virtually no activity by civil society organisations (CSOs) in the region, the need to engage multiple stakeholders involved in the decision-making process on health workforce migration and international recruitment, lack of coordinated and comprehensive data on health personnel mobility, weak national capacity to deal with health workforce issues, lack of shared understanding between stakeholders, lack of inter-country cooperation in exchanging data, and lack of proper mechanisms for sharing good practices to better manage health worker mobility [34]. The silent voice of CSOs since the adoption of the Code is noteworthy. Civil society was part and parcel of the negotiations for the Code from the outset, throughout the entire process and up to the last minute when the Code was unanimously adopted at the World Health Assembly. The CSO voice has gone silent in recent years, however, partly because funding for further CSO engagement on the Code has dried up. Without that voice, there is no one to whip countries and governments into action on the Code. A strong finding was that the Code content was not well known in the countries [34]. Strong CSO action would have ensured proper dissemination and local interpretation of the Code.

The Code is relevant and has the potential to spur action on virtually all aspects of the HRH challenges in the developing world. Nevertheless, action has been lacking on both the part of governments and CSOs.

## Discussion

The analyses of Code implementation in the European and ESA region indicate stark differences between these regions. In Europe, Code implementation and its underlying norms have been effectively addressed. Most countries are aware about the Code, and have a designated authority in place that monitors the different elements of the Code. In 2013, most of them also submitted timely reports to the World Health Assembly regarding the monitoring of Code implementation by its member states. The WHO Regional Office for Europe has offered consistent policy advice and leadership to keep the Code relevant and under attention of its member states [36]. The *EU Joint Action Health Workforce Planning and Forecasting*, a 36-month project funded by the European Commission with the objective to provide a platform for collaboration and exchange between Member States to support them to prepare the future of the health workforce, has concluded that “*The principles of the Code are also relevant within the free movement zone of the EU*” [37]. They suggest retention measures, circular migration, and better use of EU cohesion policies and the European Social Fund as policy options to mitigate unbalanced health workforce mobility within the European Region.

In addition, a vibrant coalition of civil society (to a considerable extent also financed by the European Commission), academic institutions, professional associations, and labour unions ensures that the governance of HRH migration is addressed and remains on the policy agenda. The inter-sectoral approach with involvement of multiple actors as promoted in the Code is taking place in a number of European countries. Hence, the Code remains relevant for policy guidance within the EU. However, due to the financial crisis and related austerity measures, employment opportunities for the European health workforce have diminished. There has been less recruitment from outside the European region, and more mobility of health workers between European member states. Migration mostly takes place from eastern and southern European countries to those in North and Western Europe [38]. This migration is mainly governed by European policies on the free market mobility of goods, services, and labour within the union. The European economic governance framework, the so called ‘European semester’ provides guidance for the budgetary and fiscal space that the countries have commonly agreed upon. This economic framework also offers recommendations for reforming their health system, although this remains ultimately the responsibility and competency of the member state itself. The Code, in principle more tailored to addressing imbalances and ethical considerations considering health systems development between high- and low- and middle-income countries, could also be used to mitigate this intra-European mobility, if slightly adjusted.

The ESA region offers a contrasting picture. The Code is still relevant in addressing health workforce migration, but its implementation has been far from effective. Research has indicated that the number of African physicians into the United States workforce continues to increase substantially despite the adoption of the Code [39]. The absence of health workers in Sierra Leone and Liberia due to international migration was one of the key factors undermining an effective response by the health authorities to the Ebola epidemic [40]. However, African countries have not been able to use the Code as a negotiating tool in health diplomacy to pursue their own policy interests as northern countries seem to prefer using development aid to address health worker issues rather than bilateral agreements [34]. There is a perception that these African interests are not taken seriously by the global health community, including most of the ‘donor’ countries in the North. Indeed, promises and pledges on funding for health systems strengthening have not been met over the last years [41].

Additionally, weak political leadership, limited institutional capacity, and a silenced civil society have all played a role in failing to take the principles of the Code forward. However, poor dissemination and scarcity of resources might also explain, to a certain extent, why uptake of the Code has been hampered. In contrast to the EU, where there are several inter-governmental, research, and civil society projects funded in the field of health workforce mobility, this is hardly the case in the ESA region. Additional resources could advance dissemination and advocacy amongst African policymakers to implement the Code’s articles.

Perhaps there are simply more urgent issues to address than mitigating the migration of health workers. Further, in the short run, it might even be beneficial to have migrated health workers sending their remittances home so that their families can cover basic economic needs. The long term objective of building a national health system, often in settings where institutional governance arrangements are fragile, might not be the main priority for many ESA governments, hence the disinterest to implement, monitor, and report on the Code.

There are other issues that impede the effectiveness of the Code. “Active recruitment” (article 5.1) is not further explained, allowing space to interpretation and thus confusion as to what is considered “ethical” and what is not [39]. A second assessment is that the Code lacks an enabling governance structure supported by a sustainable financing mechanism for cost-sharing and reimbursing of resource-poor countries for the mobility and loss of their public workforce. During the negotiations on the Code, low-income and emerging market countries recognized that high-income states would simply not agree to more binding provisions on financial support to developing countries. As the Code is a living document, this situation is not carved in stone however, and one could imagine a meaningful discourse on compensation in the future [11].

A policy proposal has been made to recommend a global fee-supported system similar to that employed by UNITAID. This Global Health Resource Fund would basically use a dynamic fee structure that would oblige high-income countries and private sector actors engaged in the recruitment of resource-poor country health workers to contribute with funds earmarked for health systems strengthening and employment in the public sector. This fund would build upon the existing efforts of a health systems funding platform by WHO, the World Bank, the Global Fund, and the Gavi alliance [42]. This proposal matches well with current suggestions for an international health systems fund [43] and the resolution by WHO’s Executive Board Special Session on Ebola in January 2015 that called for “*the establishment of a more extensive global, public health reserve workforce*” [44].

Finally, the governance of HRH migration has become more complex over the years, as it is now at the nexus of wider global policy initiatives and debates. The “*migration of health professionals is at the junction of the right to mobility, right to health and the right to decent work. It is about finding an acceptable compromise between the rights and obligations of migrant workers, employers and governments based on sound research findings*” [45]. A key challenge is the coordination of responses within the different multilateral organisations that are involved in the multifaceted arena of HRH migration. It is for this reason that multilateral organisations share the view that the Code is unlikely to become a binding tool in the future. Nevertheless, one should explore broader public policy coordination affecting migration. This would include, amongst others, policy coherence with the International Labour Organization’s Multilateral Framework on Labour Migration. It is, in addition, necessary to make HRH migration an issue within the post-2015 development agenda, and in the debate on the role of global trade agreements in the quest for development. Global and regional trade agreements are likely to increase (temporary) labour migration. Therefore, there are many remaining questions about the global and shared responsibility for humans to have a universal right to access health services by skilled health workers. This leads to the following question: what role can a global alliance like GHWA play with respect to the monitoring of the Code, other codes, and global commitments to keep HRH migration on global policy agendas across a range of institutional policy domains? When the relevance and effectiveness of the Code are discussed at the sixty-eighth World Health Assembly, and in relation to an upcoming Global HRH strategy, it seems vital to also discuss the necessary source and forms of global institutional leadership needed to refocus global attention on urgently needed HRH development and governance of health worker migration [45].

## Conclusions

When it comes to the relevance and effectiveness of the Code in the European and ESA regions, the picture is ambiguous. In a number of European countries the Code is effectively implemented, partly due to a dynamic civil society engagement. The financial crisis, the related austerity agenda, and the internal European policy context have made the Code even more relevant within the EU in recent years. Conversely, in the ESA region, the Code remains very relevant due to the high attrition rate of health workers migrating abroad. The Code is, however, far from being effectively implemented, mainly because policymakers and civil society do not think the Code brings many benefits. Hence, it does not have a high priority for the governments and societies in the region. There are limited resources for dissemination, advocacy, and policy support to implement the Code. The non-binding character and lack of compensation have led to a somewhat similar fate for the global Code as the bilateral and regional Codes of practice that were created over a decade ago. Solutions to overcome this situation would be to further clarify certain definitions within the Code and to develop a governance structure and a sustainable, binding financing system to reimburse countries for health workforce losses due to migration. Likewise, there is a need to address the governance of HRH migration within the context of global international labour migration frameworks, the sustainable development agenda, and the development of global and regional free trade agreements. A human rights-based approach, focusing on universal access to health care and health equity, should underpin such a global governance regime.

## Abbreviations

CSOs: civil society organisations
ESA: Eastern and Southern Africa
GHWA: global health workforce alliance
HRH: human resources for health
WHO: World Health Organization

## Footnotes

1 Sixteen countries reported changes to health worker pay, almost all in direct response to the crisis (Austria, Cyprus, Denmark, Greece, Iceland, Ireland, Italy, Latvia, Lithuania, Montenegro, Portugal, Romania, Serbia, Slovenia, Spain, United Kingdom). In some countries, especially those with economic adjustment programmes, pay cuts have been substantial.

2 The *Action Plan for the EU Health Workforce* is in fact an annex to the *Commission’s Communication towards a Job-Rich Recovery*, which sets out a range of measures to encourage employment within the Europe 2020 framework for smart, sustainable, and inclusive growth.

3 Free mobility of workers and services within the EU internal market is an economic imperative and a civil right enshrined in the Lisbon Treaty. The EU can increasingly be seen as a single labour market for health workers. It should also be seen as a protected market, given that Directive 2005/36/EC on Recognition of Professional Qualifications gives health workers from the European Economic Area (EEA) easier access to employment than their non-EEA counterparts.

4 Health Workers for All and All for Health Workers is a partnership connecting civil society organizations in Belgium, Germany, Italy, the Netherlands, Poland, Romania, Spain, and the United Kingdom.
